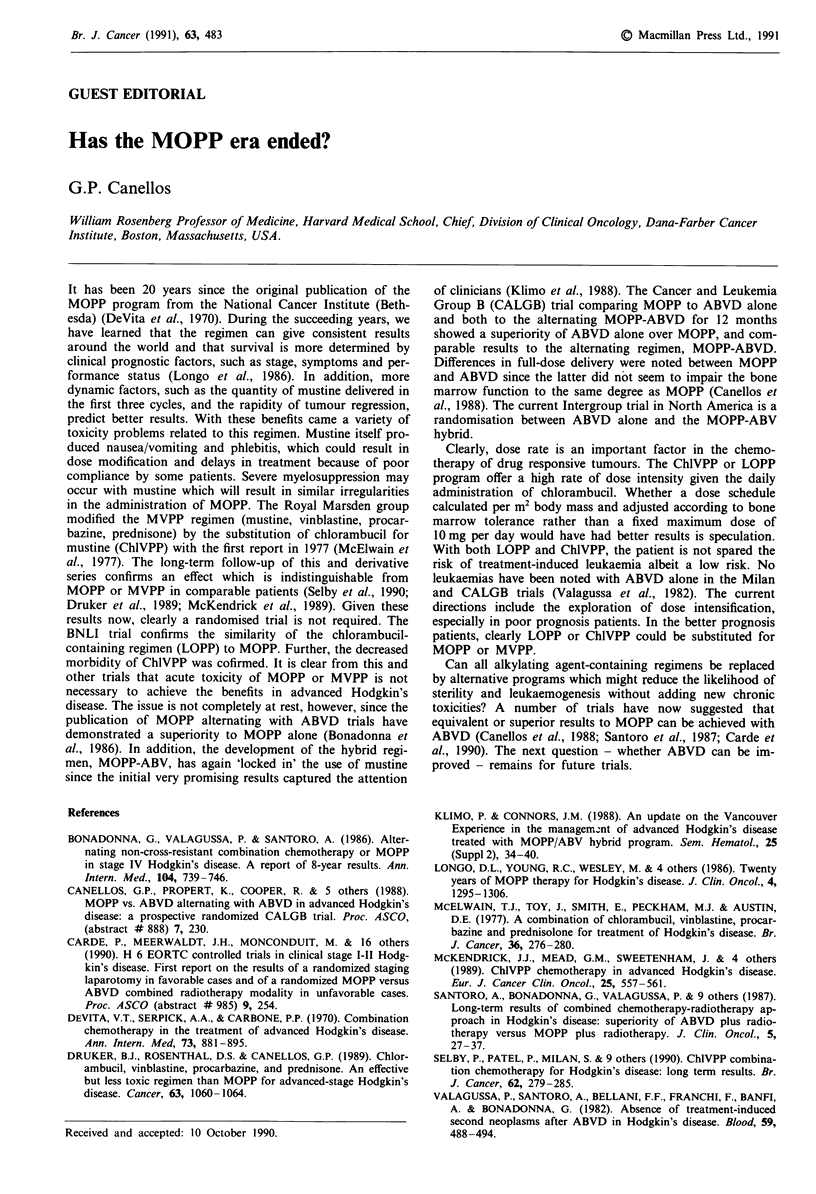# Has the MOPP era ended?

**DOI:** 10.1038/bjc.1991.115

**Published:** 1991-04

**Authors:** G. P. Canellos


*Br. J. Cancer* (1991), **63**, 483 © Macmillan Press Ltd., 1991

GUEST EDITORIAL

### Has the MOPP era ended?

G.P. Canellos

*William Rosenberg Professor of Medicine, Harvard Medical School, Chief, Division of Clinical Oncology, Dana-Farber Cancer Institute, Boston, Massachusetts, USA.*

It has been 20 years since the original publication of the MOPP program from the National Cancer Institute (Bethesda) (DeVita *et al.*, 1970). During the succeeding years, we have learned that the regimen can give consistent results around the world and that survival is more determined by clinical prognostic factors, such as stage, symptoms and performance status (Longo *et al.*, 1986). In addition, more dynamic factors, such as the quantity of mustine delivered in the first three cycles, and the rapidity of tumour regression, predict better results. With these benefits came a variety of toxicity problems related to this regimen. Mustine itself produced nausea/vomiting and phlebitis, which could result in dose modification and delays in treatment because of poor compliance by some patients. Severe myelosuppression may occur with mustine which will result in similar irregularities in the administration of MOPP. The Royal Marsden group modified the MVPP regimen (mustine, vinblastine, procarbazine, prednisone) by the substitution of chlorambucil for mustine (ChlVPP) with the first report in 1977 (McElwain *et al.*, 1977). The long-term follow-up of this and derivative series confirms an effect which is indistinguishable from MOPP or MVPP in comparable patients (Selby *et al.*, 1990; Druker *et al.*, 1989; McKendrick *et al.*, 1989). Given these results now, clearly a randomised trial is not required. The BNLI trial confirms the similarity of the chlorambucil-containing regimen (LOPP) to MOPP. Further, the decreased morbidity of ChlVPP was cofirmed. It is clear from this and other trials that acute toxicity of MOPP or MVPP is not necessary to achieve the benefits in advanced Hodgkin's disease. The issue is not completely at rest, however, since the publication of MOPP alternating with ABVD trials have demonstrated a superiority to MOPP alone (Bonadonna *et al.*, 1986). In addition, the development of the hybrid regimen, MOPP-ABV, has again 'locked in' the use of mustine since the initial very promising results captured the attention of clinicians (Klimo *et al.*, 1988). The Cancer and Leukemia Group B (CALGB) trial comparing MOPP to ABVD alone and both to the alternating MOPP-ABVD for 12 months showed a superiority of ABVD alone over MOPP, and comparable results to the alternating regimen, MOPP-ABVD. Differences in full-dose delivery were noted between MOPP and ABVD since the latter did not seem to impair the bone marrow function to the same degree as MOPP (Canellos *et al.*, 1988). The current Intergroup trial in North America is a randomisation between ABVD alone and the MOPP-ABV hybrid.

Clearly, dose rate is an important factor in the chemotherapy of drug responsive tumours. The ChlVPP or LOPP program offer a high rate of dose intensity given the daily administration of chlorambucil. Whether a dose schedule calculated per $m^2$ body mass and adjusted according to bone marrow tolerance rather than a fixed maximum dose of 10 mg per day would have had better results is speculation. With both LOPP and ChlVPP, the patient is not spared the risk of treatment-induced leukaemia albeit a low risk. No leukaemias have been noted with ABVD alone in the Milan and CALGB trials (Valagussa *et al.*, 1982). The current directions include the exploration of dose intensification, especially in poor prognosis patients. In the better prognosis patients, clearly LOPP or ChlVPP could be substituted for MOPP or MVPP.

Can all alkylating agent-containing regimens be replaced by alternative programs which might reduce the likelihood of sterility and leukaemogenesis without adding new chronic toxicities? A number of trials have now suggested that equivalent or superior results to MOPP can be achieved with ABVD (Canellos *et al.*, 1988; Santoro *et al.*, 1987; Carde *et al.*, 1990). The next question – whether ABVD can be improved – remains for future trials.

### References

BONADONNA, G., VALAGUSSA, P. & SANTORO, A. (1986). Alternating non-cross-resistant combination chemotherapy or MOPP in stage IV Hodgkin's disease. A report of 8-year results. *Ann. Intern. Med.*, **104**, 739-746.

CANELLOS, G.P., PROPERT, K., COOPER, R. & 5 others (1988). MOPP vs. ABVD alternating with ABVD in advanced Hodgkin's disease: a prospective randomized CALGB trial. *Proc. ASCO*, (abstract # 888) **7**, 230.

CARDE, P., MEERWALDT, J.H., MONCONDUIT, M. & 16 others (1990). H 6 EORTC controlled trials in clinical stage I-II Hodgkin's disease. First report on the results of a randomized staging laparotomy in favorable cases and of a randomized MOPP versus ABVD combined radiotherapy modality in unfavorable cases. *Proc. ASCO* (abstract # 985) **9**, 254.

DEVITA, V.T., SERPICK, A.A., & CARBONE, P.P. (1970). Combination chemotherapy in the treatment of advanced Hodgkin's disease. *Ann. Intern. Med*, **73**, 881-895.

DRUKER, B.J., ROSENTHAL, D.S. & CANELLOS, G.P. (1989). Chlorambucil, vinblastine, procarbazine, and prednisone. An effective but less toxic regimen than MOPP for advanced-stage Hodgkin's disease. *Cancer*, **63**, 1060-1064.

KLIMO, P. & CONNORS, J.M. (1988). An update on the Vancouver Experience in the management of advanced Hodgkin's disease treated with MOPP/ABV hybrid program. *Sem. Hematol.*, **25** (Suppl 2), 34-40.

LONGO, D.L., YOUNG, R.C., WESLEY, M. & 4 others (1986). Twenty years of MOPP therapy for Hodgkin's disease. *J. Clin. Oncol.*, **4**, 1295-1306.

McELWAIN, T.J., TOY, J., SMITH, E., PECKHAM, M.J. & AUSTIN, D.E. (1977). A combination of chlorambucil, vinblastine, procarbazine and prednisolone for treatment of Hodgkin's disease. *Br. J. Cancer*, **36**, 276-280.

McKENDRICK, J.J., MEAD, G.M., SWEETENHAM, J. & 4 others (1989). ChlVPP chemotherapy in advanced Hodgkin's disease. *Eur. J. Cancer Clin. Oncol.*, **25**, 557-561.

SANTORO, A., BONADONNA, G., VALAGUSSA, P. & 9 others (1987). Long-term results of combined chemotherapy-radiotherapy approach in Hodgkin's disease: superiority of ABVD plus radiotherapy versus MOPP plus radiotherapy. *J. Clin. Oncol.*, **5**, 27-37.

SELBY, P., PATEL, P., MILAN, S. & 9 others (1990). ChlVPP combination chemotherapy for Hodgkin's disease: long term results. *Br. J. Cancer*, **62**, 279-285.

VALAGUSSA, P., SANTORO, A., BELLANI, F.F., FRANCHI, F., BANFI, A. & BONADONNA, G. (1982). Absence of treatment-induced second neoplasms after ABVD in Hodgkin's disease. *Blood*, **59**, 488-494.

Received and accepted: 10 October 1990.